# Identification of *ATP2C1* mutations in the patients of Hailey-Hailey disease

**DOI:** 10.1186/s12881-020-01056-4

**Published:** 2020-06-01

**Authors:** Xiaoli Li, Dingwei Zhang, Jiahui Ding, Li Li, Zhenghui Wang

**Affiliations:** 1 Department of Dermatology, The Second Affiliated Hospital, Xi’an Jiaotong University, Xi’an, 710004 China; 2 Department of Otolaryngology-Head and Neck Surgery, The Second Affiliated Hospital, Xi’an Jiaotong University, NO. 157 Xi Wu Road, Xi’an, 710004 Shaan’xi Province China

**Keywords:** Familial benign chronic pemphigus, *ATP2C1*, Gene mutation, p63

## Abstract

**Background:**

Familial benign chronic pemphigus, also known as Hailey-Hailey disease (HHD), is a clinically rare bullous Dermatosis. However the mechanism has not been clarified. The study aim to detect novel mutations in exons of *ATP2C1* gene in HHD patients; to explore the possible mechnism of HHD pathogenesis by examining the expression profile of hSPCA1, miR-203, p63, Notch1 and HKII proteins in the skin lesions of HHD patients.

**Methods:**

Genomic DNA was extracted from peripheral blood of HHD patients. All exons of *ATP2C1* gene in HHD patients were amplified by PCR and the products were purified and sequenced. All related signaling proteins of interest were stained by using skin lesion tissues from HHD patients and miR-203 levels were also determined.

**Results:**

One synonymous mutation c.G2598A (in exon 26), one nonsense mutation c.C635A and two missense mutations c.C1286A (p.A429D) and c. A1931G (p. D644G) were identified. The nonsense mutation changed codon UCG to stop codon UAG, causing a premature polypeptide chain of the functional region A. The two missense mutations were located in the region P (phosphorylation region) and the Mn binding site of hSPCA1. The level of hSPCA1 was significantly decreased in HHD patients compared to the normal human controls, accompanied by an increase of miR-203 level and a decrease of p63 and HKII levels.

**Conclusion:**

In our study, we found four mutations in HHD. Meanwhile we found increase of miR-203 level and a decrease of p63 and HKII levels. In addition, Notch1, which was negatively regulated p63, is downregulated. These factors may be involved in the signaling pathways of HHD pathogenesis. Our data showed that both p63 and miR-203 may have significant regulatory effects on Notch1 in the skin.

## Background

Familial benign chronic pemphigus, also known as Hailey-Hailey disease (HHD), is a clinically rare bullous Dermatosis, and mainly manifested as erosion, erythema, accompanied with blisters and pimples. The incidence of this disease is approximately 0.002% [1], and has no significant difference between men and women [2]. HHD is currently not curable, and symptomatic treatment is the main strategy taken so far to reduce symptoms and prevent disease recurrence [3–6], using cortisol to reduce inflammation [7], and using topical vitamins [8, 9] to promote the differentiation of epidermal keratinocytes and regulate intercellular Ca^2+^ concentrations [10, 11].

HHD is caused by mutations in the *ATP2C1* gene. *ATP2C1* is expressed in the skin as well as the brain, skeletal muscle, placenta, heart, and lungs. In humans, *ATP2C1* mutations rarely cause skin tumors, squamous cell carcinoma and basal cell carcinoma [12, 13] in HHD lesions. Liver failure with HHD was ever reported [14]. There are also some cases that have emotional disorders have no desire in almost all activities [13]. It is believed that HHD development is due to insufficient gene dose of *ATP2C1*. The protein level of hSPCA1 (the human secretory pathway Ca^2+^/Mn^2+^ − ATPase protein 1, hSPCA1) encoded by the *ATP2C1* in the Golgi apparatus is strikingly decreased, resulting in an increase in the intracellular Ca^2+^ concentration. As an important messenger, Ca^2+^ has significant impact on the maturation and differentiation of keratinocytes. Increased Ca^2+^ concentration eventually destroy cell-to-cell connections [1].

In this study, we searched for novel mutations of *ATP2C1* by sequencing the gene from 2 different HHD pedigrees and 2 sporadic cases. We analyzed the impact of these mutations on the structure and function of hSPCA1 by using bioinformatics tool. Meanwhile the expression levels of hSPCA1, miR-203, p63, Notch1, and HKII in the skin lesion tissues of HHD patients were examined.

## Methods

### Patients

The study subjects were HHD patients enrolled in the dermatology clinic of the Second Affiliated Hospital of Xi’an Jiaotong University, and pathological biopsy was performed on the patient’s typical skin lesions to further confirm the diagnosis. All procedures were approved by the Institutional Human Experiment and Ethics Committee of the Second Hospital of Xi’an Jiaotong University. The samples from 2 patients in pedigree I, II and 2 sporadic patients were collected. The patient’s information such as gender, age, and skin lesion performance, time of onset, and family history was collected.

### HE staining

Tissues were selected from a typical site of the patient’s skin lesions following a biopsy under local anesthesia. The removed tissues were placed in 4% formaldehyde solution and fixed at 4 °C for > 4 h; the tissues were then rinsed under water for 10 min and then dehydrated in a biological tissue dehydrator following standard procedures. The tissues were then immediately put into 60 °C paraffin, and poured into the embedding box. After the paraffin is completely cooled down, the tissue was sectioned with thickness of 5 μm. The tissue sections were deparaffined following to standard procedures [15].

### DNA extraction

A total of 2 ml of peripheral venous blood was drawn from 2 patients with HHD from two different pedigrees and 2 sporadic patients following to the patient’s consent. As controls, 2 ml of peripheral blood were collected from the patient’s healthy family members and 100 normal people who were not biological related. The blood was collected in a 2% Ethylene Diamine Tetraacetic Acid (EDTA) anticoagulant tube. The DNA was immediately extracted or stored in a − 20 °C refrigerator. DNA extraction from peripheral blood was performed according to the following protocols: 200 μl of proteinase K solution was added to 200 μl of fresh or thawed blood and mixed well, and then 200 μl of binding solution (CB) was added and mixed thoroughly by vortexing, and incubated at 70 °C for 10 min; 100 μl of isopropanol was added after the samples cooled down, vortexed immediately to mix thoroughly, and then put it into the AC column, centrifuged at 13,000 rpm for 60 s, and then discarded the flow-through in the collection tube; the inhibitor remover (IR) and the rinse solution (WB) were added sequentially, centrifuged at 12,000 rpm for 30 s and discarded the flow-through. 100 μl of pre-warmed elution buffer EB was added onto the center of the adsorption membrane, and then centrifuged at 12,000 rpm to collect DNA solution; the DNA product was stored at 4 °C, or at − 20 °C for long-term use.

### *ATP2C1* primers and PCR reactions

Primer sequences were designed and verified using Primer 5.0. All upstream and downstream primers cover some intron sequences on both sides of the exon. Primer synthesis and purification of PCR product were completed by Beijing Aoke Dingsheng Biotechnology Co., Ltd. PCR reaction: 2 × Taq Master MIX, 25 μl; DNA template, 5 μl; forward primer, 1 μl; reverse primer 1 μl; double distilled water, 18 μl; total volume, 50 μl. PCR protocol was summarized in Table 1. Protocol I was used for exon 1, protocol III was used for exon 5, 7, 12, 27 and 28 and protocol II was used for other exons [15]. The sequencing data of each exon was read by Chromas 2.4 and aligned with the original sequence of human *ATP2C1* gene.

**Table 1:** PCR protocol

94 °C 5 min					
I	94 °C 45 s	60 °C 45 s	72 °C 45 s	2×	1
	94 °C 45 s	59 °C 45 s	72 °C 45 s	2×	
	94 °C 45 s	58 °C 45 s	72 °C 45 s	2×	
	94 °C 45 s	57 °C 45 s	72 °C 45 s	2×	
	94 °C 45 s	56 °C 45 s	72 °C 45 s	2×	
	94 °C 45 s	55 °C 45 s	72 °C 45 s	20×	
II	94 °C 45 s	55 °C 45 s	72 °C 45 s	32×	Other exon
III	94 °C 30s	54 °C 30s	72 °C 30s	30×	5, 7, 12, 27, 28

### IHC staining

The skin tissues collected from patients with HHD were cut into thickness of 4–5 μm by using a paraffin microtome. The sections were deparaffined and rehydrated following to standard procedures. Hydrogen peroxide (0.3%) was added onto the sections to block endogenous peroxidase. The sections were then incubated with 0.1% saponin for 30 min; 10% normal goat serum was added onto the sections and incubated at 37 °C for 30 min to cover non-specific sites; the blocking serum was then discarded and the primary antibody (hSPCA1, p63, Notch1, HKII; 1:50 dilution) was added and incubated at 4 °C for 24–72 h; secondary antibodies (goat anti-rabbit or goat anti-mouse) labeled with horseradish peroxidase was added and incubated at 37 °C for 30 ~ 40 min and then washed with PBS for 3 times, 5 ~ 10 min each time [15].

### Real time-PCR

Total RNA was extracted and cDNA was synthesized according to the kit instructions. The primers were synthesized by Beijing Aoke Dingsheng Biotechnology Co., Ltd. according to the following sequences. miR-203: Forward 5′- ttgagttagggttatttttgtgt − 3 ‘; Reverse 5’-ctaacccaaccaatttttccaa − 3′. The PCR reaction: 2 × Taq Master Mix, 10 μl; cDNA, 2 μl; forward primer; 0.2 μl; reverse primer, 0.2 μl; DEPC water, 7.4 μl; total volume, 20 μl. PCR protocol: pre-denaturation, 95 °C, 3 min; denaturation, 95 °C, 12 s; annealing, 62 °C, 35 s; extension, 74 °C, 3 s; 40 cycles; extension, 74 °C, 5 min. The expression level of each gene was determined by relative quantification, and GAPDH included in the kit was used as an endogenous control. The relative expression level was determined by using the Ct value. Relative expression level = 2^-△△CT^.

## Results

### Pedigree analysis and clinical data of familial benign chronic pemphigus

#### Pedigree analysis and clinical data of pedigree I

**Fig. 1:**
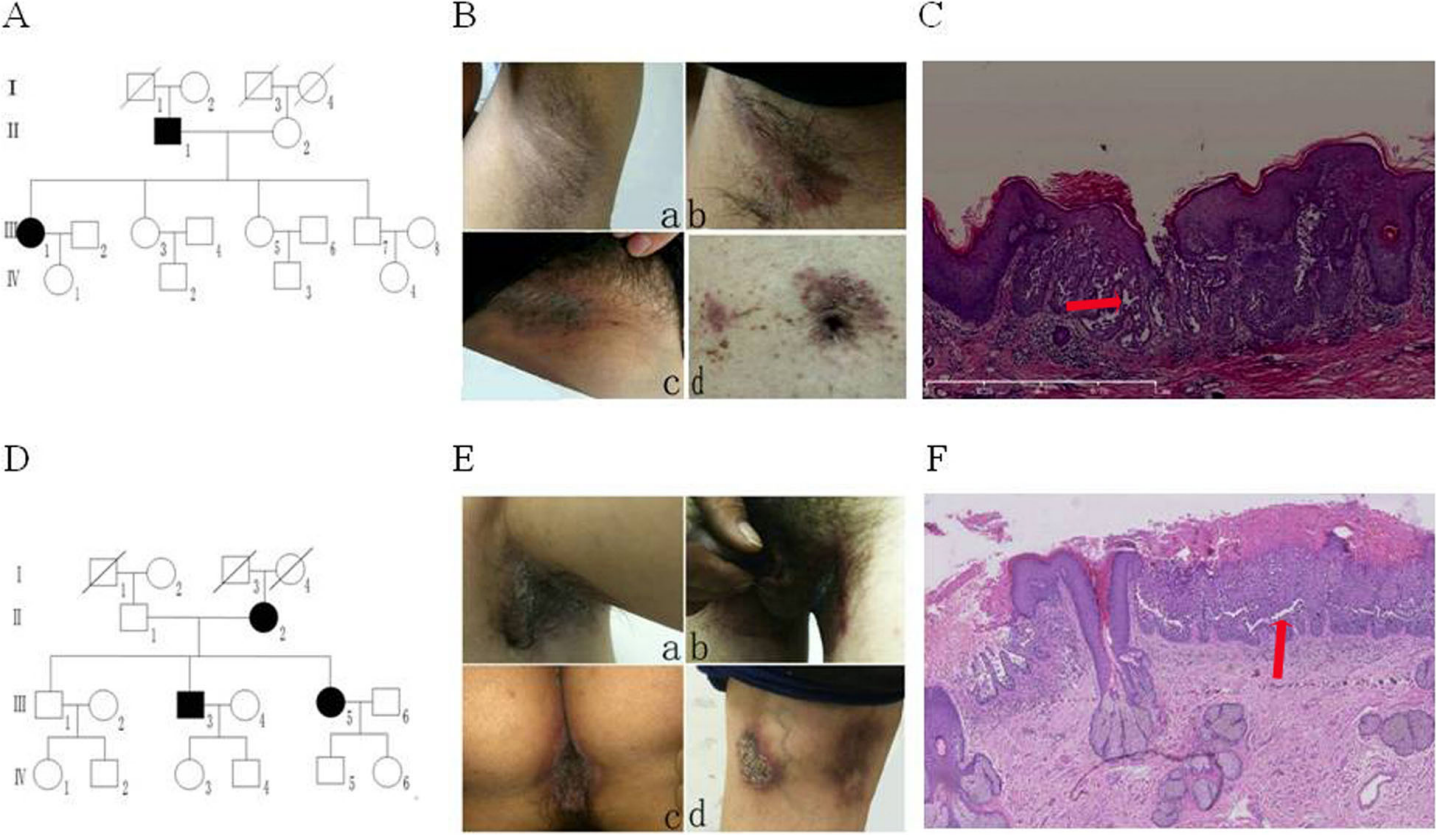
Pedigree analysis and clinical data of pedigree I and II. (A) Pedigree chart of pedigree I. (B) Clinical manifestations of the proband in pedigree I. (C) Histopathological examination of typical skin lesion for the proband in pedigree I (5×)(Arrow shows acanthosis). (D) Pedigree chart of pedigree II. (E) Clinical manifestations of the proband in pedigree II. (F) Histopathological examination of typical skin lesion for the proband in pedigree II (5×) (Arrow shows acanthosis)

Among 4 generations of the pedigree, there are 2 HHD patients (1 male and 1 female) who were present in the 2nd and 3rd generations, consistent with the inheritance pattern of autosomal dominant genetic disease (Fig. 1A). The proband is a 50 year-old woman whose parents were not biological related. Physical exam showed that she had good health condition in general, and no abnormality was found in other body systems. Clinical manifestations included erythema, blisters, erosions under bilateral axilla, left groin and umbilicus, and slight hypertrophy of skin in the left groin (Fig. 1B). Histopathological examination of typical skin lesions in the left groin revealed cracks in the epidermis and partial loss of the stratum corneum. The spines in the lower and middle layers of epidermis were loosened and cells of spinous layer were visible (Fig. 1C).

#### Pedigree analysis and clinical data of family II

Among 3 generations of the pedigree, there were 3 patients, one male and two females. The patients were present in two consecutive generations II and III, consistent with the inheritance pattern of autosomal dominant genetic disease (Fig. 1D). The proband was a 43 year-old male whose parents were not blood related. Physical exam showed that he had good general health condition without abnormality found in other body system. Clinical manifestations included flaky erythematous papule under the left armpit and left groin, perianal area, left popliteal fossa, adjacent popliteal fossa, obvious erosion in addition to some erythema (Fig. 1E). The left axillary skin lesions were taken for pathological examination. The results showed that the spines in the lower and middle layers of epidermis were loosened, and were overflowed on the top with red blood cells. A single layer of basal cells covered on the dermal papilla and formed a villi (Fig. 1F).

#### Clinical data of sporadic case I

**Fig. 2:**
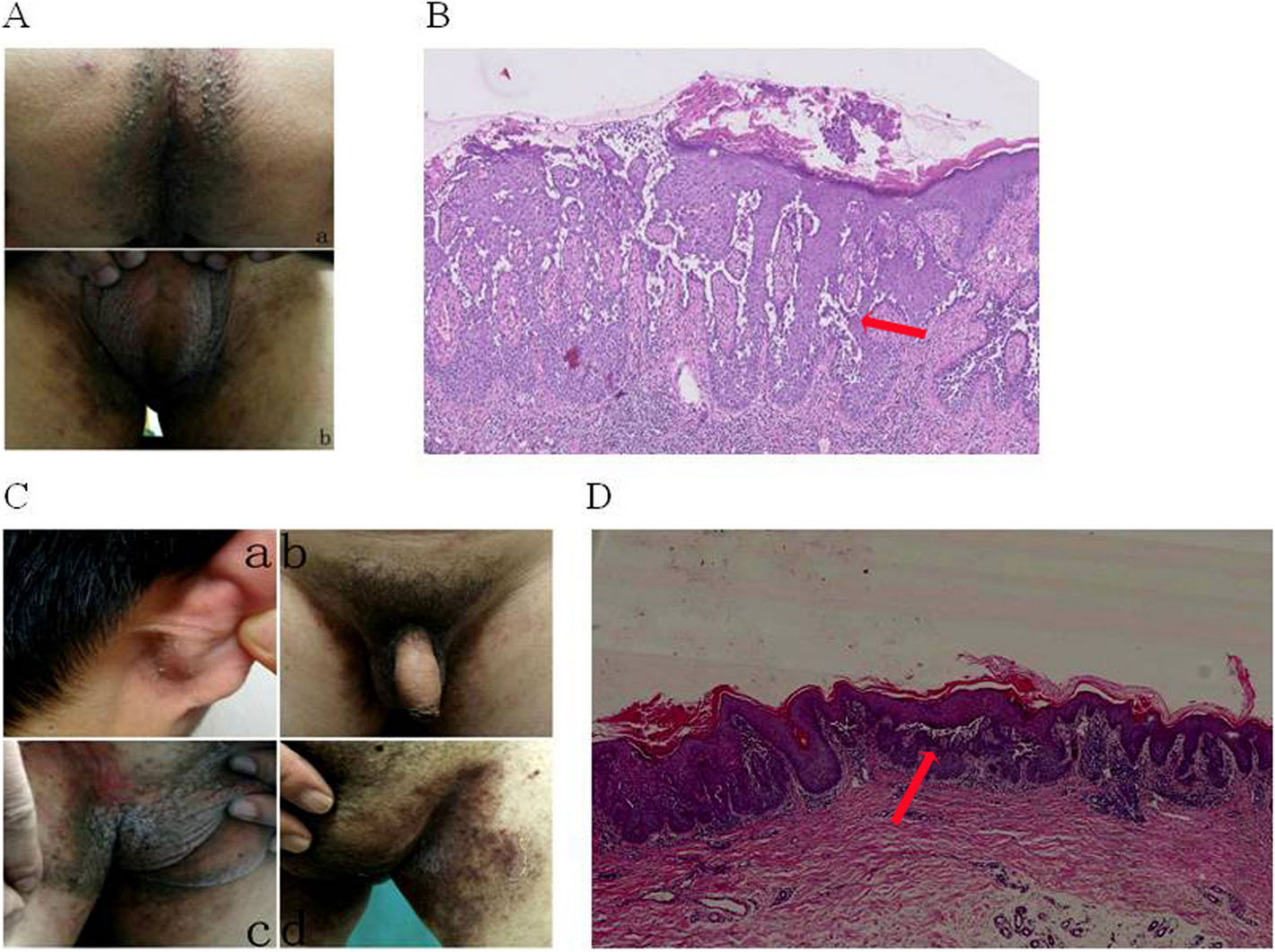
Clinical data of sporadic case I and II. (A) Clinical manifestations of sporadic case I. Skin lesions were present in the anus (a) and groin (b). (B) Histopathological examination of typical skin lesion for sporadic case I (5×) (Arrow shows acanthosis). (C) Clinical manifestations of sporadic case II. Skin lesions were present in the right back of the ear (a) and bilateral groin (b; c, left, d, right). (D) Histopathological examination of typical skin lesion for sporadic case II (5×) (Arrow shows acanthosis)

Among 3 generations of the pedigree, there was only 1 patient. No other patients in the pedigree were seen. No obvious sign of familial genetic disease was observed. The patient was a 68 year-old male whose parents were not blood related. Physical exam showed that this patient was generally in a good health condition without obviously abnormalities found in other body systems. Clinical manifestations included erythema on both sides of the groin and anus, scattered papules in addition to the anal erythema, obvious erosion and exudation in the skin lesions (Fig. 2A). A piece of skin from lesions on the left groin was taken for histological exam. The results showed that spines in the middle and lower layers of the epidermis were loosen and formed fissures in which loosening cells were presented (Fig. 2B).

#### Clinical data of sporadic cases II

Among 3 generations of the pedigree, there was 1 patient only. No other patients in the pedigree were seen. No obvious inheritance pattern of familial genetic diseases was shown. The patient was a 42 years old male whose parents were not blood related. Physical exam showed that this patient had good general health condition without other abnormalities found in other body systems. Clinical manifestations included slight erythema and desquamation on the back of right ear, erythematous papular rash on both sides of the groin, obvious erosion and exudation in addition to some erythema, and epidermal lesions on the left groin covered with crust (Fig. 2C). Histopathological examination of the typical skin lesions from the left groin showed that the spinal layer in the epidermis formed fissures in which the loosening cells were scattered around (Fig. 2D).

### Gene sequencing of *ATP2C1*

#### PCR amplification for the exons of *ATP2C1*

**Fig. 3:**
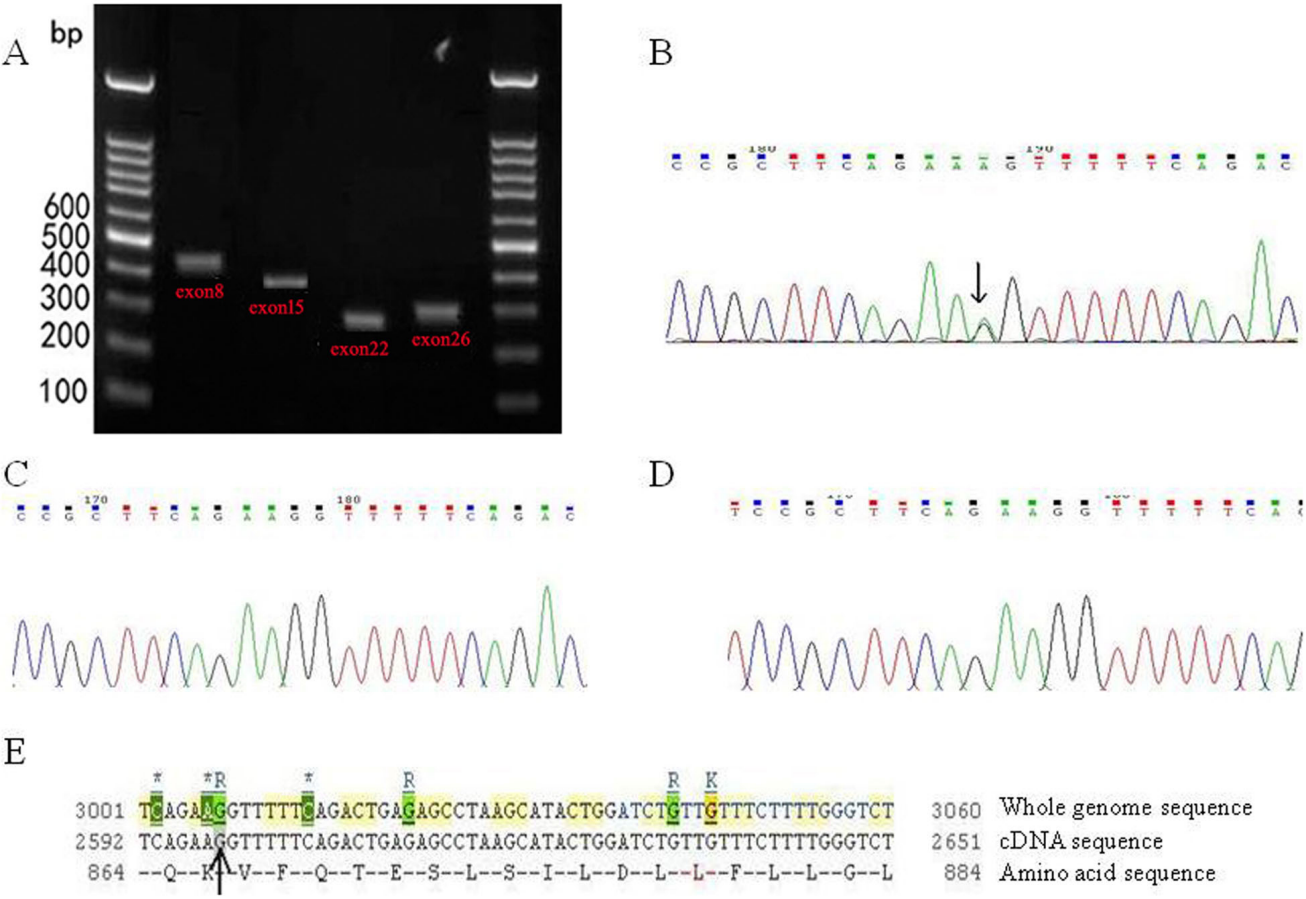
Exon 26 sequencing for the *ATP2C1* gene in pedigree I. **a** PCR amplification for exons 8, 15, 22 and 26 of *ATP2C1*. **b** Sequencing result of exon 26 for the proband in pedigree I. **c** Sequencing result of exon 26 for normal member in the same pedigree. **d** Sequencing result of exon 26 for normal individual who was not blood related. **e** A synonymous mutation c.G2598A(Bases G → A) in exon 26 of *ATP2C1* gene in the Ensemble database (indicated by arrows)

Exon 26 of *ATP2C1* in pedigree I, exon 15 of *ATP2C1* in pedigree II, exon 8 of *ATP2C1* in sporadic patient I, and exon 22 of *ATP2C1* in sporadic patient II were amplified (Fig. 3a). The sizes of PCR products were 312 bp, 383 bp, 425 bp and 298 bp respectively.

#### The sequencing result of exon 26 of *ATP2C1* in pedigree I

A synonymous mutation c.G2598A in exon 26 was found in the proband of pedigree I, which was further confirmed by reverse sequencing that showed a complementary nucleotide mutation. The mutation did not change amino acid code of lysine. We further verified this mutation was actually a nonsense mutation that was not previously reported (Fig. 3b). Such mutation was not found in the normal members of this pedigree or other people who were not blood related (Fig. 3c, d, e).

#### Sequencing results of *ATP2C1* exon 15 in pedigree II

**Fig. 4:**
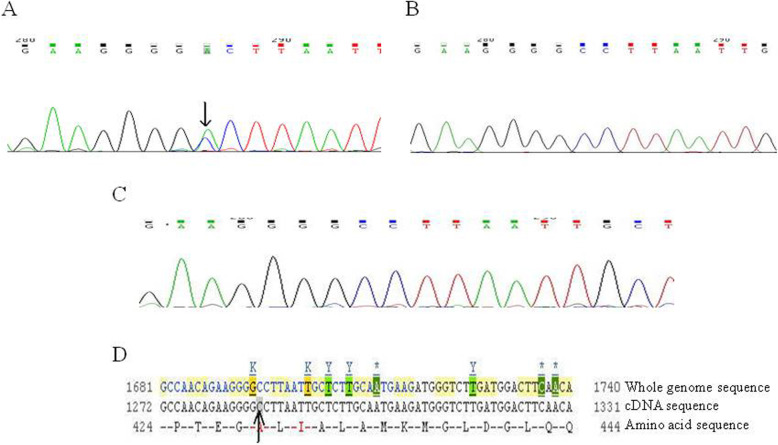
Exon 15 sequencing for the *ATP2C1* gene in pedigree II. **a** Sequencing result of exon 15 for the proband in pedigree II. **b** Sequencing result of exon 15 for normal member in the same pedigree. **c** Sequencing result of exon 15 for normal individual who was not blood related. **d** A missense mutation c.C1286A(Bases C → A) in exon 15 of *ATP2C1* gene in the Ensemble database (indicated by arrows)

A missense mutation c.C1286A was detected in the exon 15 of the proband in pedigree II, which was further confirmed by reverse sequencing that showed a complementary nucleotide mutation. This mutation changed the original alanine to aspartic acid (p.A429D). We further verified that it was actually an unreported missense mutation (Fig. 4a-d). Such mutation was not found in the normal members of this pedigree or other people who were not blood related (Fig. 4b, c).

#### Sequencing results of *ATP2C1* exon 8 in sporadic case I

**Fig. 5:**
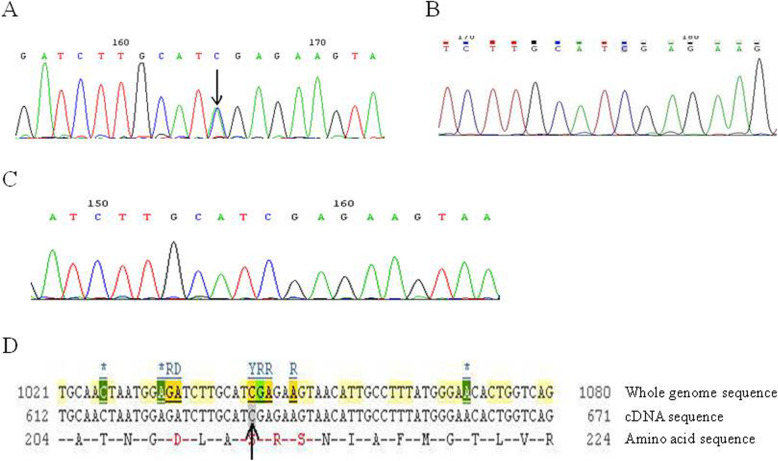
Exon 8 sequencing for the *ATP2C1* gene in sporadic case I. **a** Sequencing result of exon 8 in sporadic case I. **b** Sequencing result of exon 8 for normal member in the same pedigree. **c** Sequencing result of exon 8 for normal individual who was not blood related. **d** A nonsense mutation c.C635A(Bases C → A) in exon 8 of *ATP2C1* gene in the Ensemble database (indicated by arrows)

A nonsense mutation c.C635A in exon 8 was detected in the proband of sporadic case I. This mutation was further confirmed by reverse sequencing that showed a complementary nucleotide mutation. This mutation resulted in a premature stop codon in the polypeptide chain and was previously reported (Fig. 5a-d). Such mutation was not seen in the normal human controls (Fig. 5b, c).

#### Sequencing results of *ATP2C1* exon 22 in sporadic case II

**Fig. 6:**
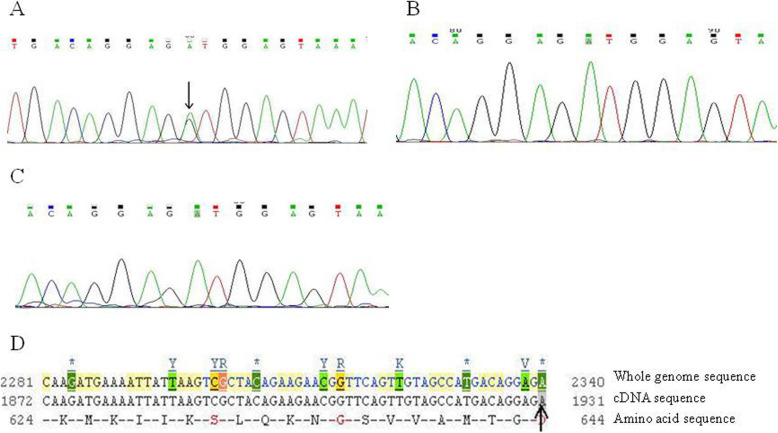
Exon 22 sequencing for the *ATP2C1* gene in sporadic case II. **a** Sequencing result of exon 22 in sporadic case II. **b** Sequencing result of exon 22 for normal member in the same pedigree. **c** Sequencing result of exon 22 for normal individual without blood relation. **d** A missense mutation c. A1931G(Bases A → G) in exon 22 of *ATP2C1* gene in the Ensemble database (indicated by arrows)

A missense mutation c. A1931G was detected in exon 22 of the proband in sporadic case II, which was further confirmed by reverse sequencing that showed a complementary nucleotide mutation. This mutation changed the original 644th aspartic acid (GAU) to be Glycine (GGU) (p. D644G). We noticed that this mutation was previously reported (Fig. 6a-d). Such mutation in the gene was not found in the normal members in the pedigree and other people who were not blood related (Fig. 6b, c).

#### IHC staining for hSPCA1

**Fig. 7:**
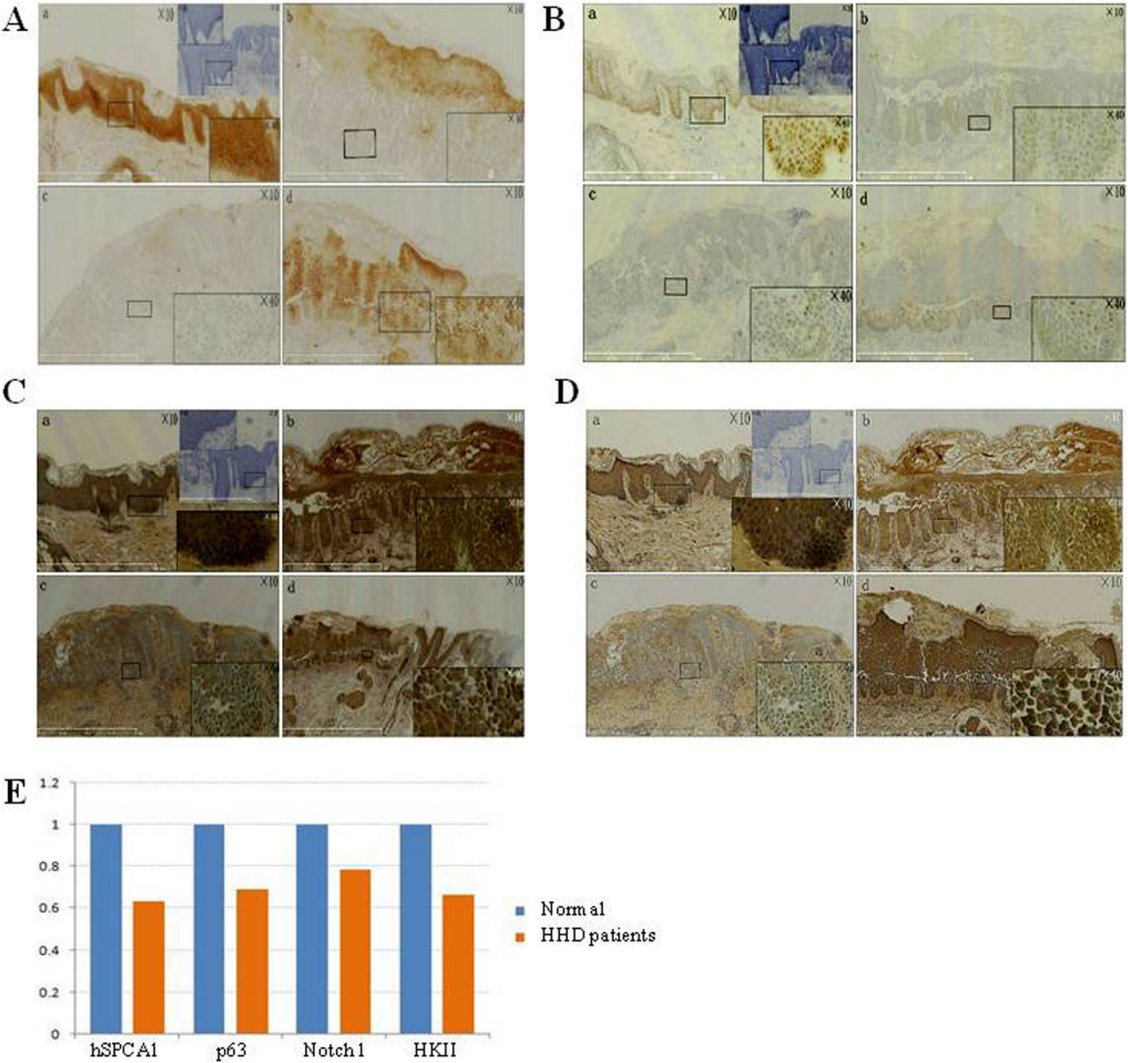
(A) IHC staining for *ATP2C1*[Negative control with PBS(a) and expression levels of hSPCA1 in HHD(b,c,d)]. (B) IHC staining for p63[Negative control with PBS(a) and p63 protein in the nucleus of HHD patients(b,c,d)]. (C) IHC staining for NotchI in normal skin (a) and HHD (b,c,d). (D) IHC staining for HKII in normal skin(a) and HHD patients(b,c,d). (E). Quantification for IHC staining signals in A ~ D

hSPCA1 is mainly expressed in the cytoplasm of keratinocytes in the epidermal layer with some on the cell membrane. Positive staining appeared as yellow or tan particles or clumps. In normal human skin tissues (Fig. 7A-a), hSPCA1showed strong positive signals, that is, a dark brown stained band in the epidermis. The negative control in which the hSPCA1 antibody was replaced with PBS (Fig. 7A-a, right corner inset) did not show any nonspecific staining. The expression levels of hSPCA1 in the skin tissues of all patients with HHD (Fig. 7A-b/c/d, E) were significantly lower than that of the positive control, especially at the typical skin lesions (acanthosis). No obvious staining signal was seen even in some local areas (Fig. 7A-c).

### IHC staining for proteins in relevant signaling pathways

#### IHC for p63

P63 is an intranuclear protein and can be stained in the nucleus of keratinocytes in the epidermal layer. In normal human skin tissues, p63 staining was strongly positive in the nucleus. In the area adjacent to the stratum corneum the nucleus gradually disappeared with differentiation, therefore, the staining is mainly present in the basal layer (Fig. 7B-a). PBS was used as a negative control, which did not show any non-specific staining (Fig. 7B-a, right corner inset). The expression level of p63 protein in the nucleus of HHD patients was significantly reduced compared to the positive control, especially in the typical lesions (Fig. 7B-b/c/d, E). No obvious staining was seen in some local areas of the tissue.

#### IHC for Notch1

Notch1 is expressed in the cytoplasm of keratinocytes in the epidermal layer. In normal human skin tissues, Notch1 was strongly stained, appeared as brown or tan particles or clumps (Fig. 7C-a). In the entire epidermal layer, Notch1 showed a even deep-stained band. In the skin tissues of all HHD patients, Notch1 signals (Fig. 7C-b/c/d, E) were weaker compared to the positive control. In contrast, the negative control by using PBS instead of Notch1 antibody did not show any non-specific staining (Fig. 7C-a, right corner inset).

#### IHC for HKII protein

HKII is expressed in the cytoplasm of the keratinocytes in the epidermal layer. In normal human skin tissues, HKII was strongly stained (Fig. 7D-a) and showed a even deep-stained band in the entire epidermal layer. In contrast, the expression level of HKII in the cytoplasm of skin lesion tissues was significantly reduced compared to the normal human positive control (Fig. 7D-b/c/d, E). The negative control by using PBS other than HKII antibody (Fig. 7D-a, right corner inset) did not show obviously non-specific staining.

### Real time PCR for miR-203

**Fig. 8:**
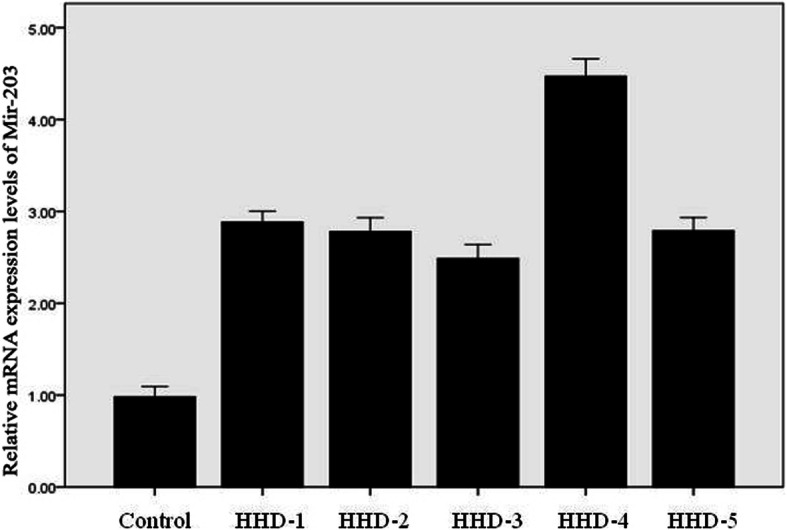
MiR-203 expression in HHD patients. Real-time PCR was performed to examine the mRNA expression levels of miR-203 in HHD patients

RNA was extracted from the skin lesion tissues of HHD patients and real-time PCR was performed to detect the expression level of miR-203 in these tissues. Normal human skin tissue was used as a control. The data showed that the levels of miR-203 in the skin lesions of HHD patients were significantly upregulated compared to that in the normal human control (Fig. 8).

## Discussion

In this study, all patients (including all pedigree cases and sporadic cases) we collected have typical clinical manifestations and histopathological characteristics. Pedigree I and pedigree II showed vertical transmission and presented in an inheritance pattern of autosomal dominant genetic disease. By using gene sequencing and exon alignment of *ATP2C1* from 4 patients, we found 4 different heterozygous mutations, including 1 synonymous mutation c.G2598A, 2 missense mutations c.C1286A and c. A1931G, and 1 nonsense mutation c.C635A.

In the patient of pedigree I, the synonymous mutation c.G2598A in exon 26 changed the codon from AAG to AAA, but did not change the amino acid (lysine). In the patient of pedigree II, the missense mutation c.C1286A (p.A429D) in exon 15 changed the alanine to aspartic acid. Protein structural analysis showed that this mutation was located in the P region (phosphorylation region), the most important functional site of hSPCA1 to which Ca^2+^/Mn^2+^ and ATP are bound. This mutation changed the primary structure of the protein and disrupted the catalytic site of ATPase, phosphorylation function and protein conformation, leading to loss of protein function and transport failure of Ca^2+^/Mn^2+^ and abnormal concentrations of intracellular Ca^2+^/Mn^2+^ in the cells.

In keratinocytes, hSPCA1 not only transports Ca^2+^, but also competitively transports one Mn^2+^ from the cytoplasm to the Golgi apparatus, which is nonreplaceable by other calcium pumps. It is known that the cells are very sensitive to Mn^2+^ concentration and a slight change of Mn^2+^ concentration may affect cell metabolism seriously [16]. Excessive intracellular Mn^2+^ may prevent Mg^2+^ binding to proteins, compromise the fidelity of DNA polymerase, and disrupt the transport function of cell membranes, thus affecting the physiological conditions of human body [17, 18]. In sporadic case I, a nonsense mutation c.C635A in exon 8 changed the serine codon UCG (position 212) to a stop codon UAG. This mutation is located in the A region of regulatory element and results in premature polypeptide chain. In addition, the mRNA with this nonsense mutation is easily degraded, reducing the expression level of truncated hSPCA1 that lacks important structures in the calcium pump. In sporadic case II, a missense mutation c. A1931G in exon 22 changed aspartate (GAU, at position 644) to glycine (GGU) (p. D644G). This mutation is located at the Mn^2+^ binding site of hSPCA1 [19] and disrupts the binding of hSPCA1 to Mn^2+^, thus interfere the metabolism of intracellular Mn^2+^ [20], affect the activity of DNA polymerase and the transport function of cell membranes, and increase the potential of mutation in other genes [21]. hSPCA1 is highly expressed in the skin and kidney tissues, localized in cytoplasmic Golgi apparatus in the cells. The clinical symptoms of *ATP2C1* mutation are mainly limited to the skin of the patients. Therefore, we chose skin lesions to investigate dysregulated signaling pathways in HHD patients. Immunohistochemistry data showed that the expression levels of hSPCA1 in the skin tissues of all HHD patients was significantly downregulated compared to normal human controls.

MiR-203 is a family member of tumor suppressor genes. It is a type of short, non-coding single-stranded miRNA [22]. MiR-203 has the highest abundance in the skin, and is exclusively expressed in keratinocytes, indicating that it may be a key to various biological functions of keratinocytes [23]. When keratinocytes treated with high concentration of Ca^2+^, miR-203 was upregulated > 3-fold [23, 24]. In the keratinocytes of HHD patients, Ca^2+^ concentration is strikingly increased due to the dysfunction of the calcium pump. In our study, miR-203 in the skin lesions of patients showed a significant upregulation.

The transcription factor p63 is a member of the p53 tumor suppressor family [25] and plays an important role in the regulation of epidermal growth, keratinocyte differentiation, cell adhesion and cell migration [26]. During keratinization, the expression level of *ATP2C1* is strikingly decreased, so does p63. Numerous studies have shown that p63 regulates downstream target genes and also plays roles in a variety of intracellular signaling pathways [27, 28]. p63 and Notch1, two important factors that regulate cell proliferation and differentiation, cell adhesion and other functions in keratinocytes, are negatively regulated to each other [23]. Mature miR-203 binds to p63 3′-UTR and downregulate p63 [29]. In animal experiments, miR-203 affected the “stemness” of mouse skin stem cells by targeting p63 expression, thus enhancing the differentiation capacity of basal cells [30, 31]. MiR-203 can also inhibit Notch1 expression by downregulating ligand Jagged1 in the Notch1 pathway [32, 33]. In our study we found p63 was significantly downregulated compared to the normal human controls. From previous studies and ours, it is believed that increased intracellular Ca^2+^ concentration is actually accompanied by upregulation of miR-203 and downregulation of p63. We also found that the expression level of Notch1, which is negatively regulated by p63, only showed a slight decrease. Our data suggest that p63 and miR-203 are both associated with Notch1 in the skin lesions.

Hexokinase is a key enzyme in the first step of glycolytic pathway, catalyzing glucose into glucose 6-phosphate. In human cells, hexokinase has four subtypes (I-IV), of which HKII is the one present in human keratinocytes and regulates metabolism of glucose and lactate. Therefore, inhibiting HKII activity can effectively block sugar metabolism. HKII gene is a target gene of p63, and p63 downregulation can reduce 80% of HKII mRNA. In keratinocytes, the p63-HKII axis is an important pathway regulating cell metabolism and proliferation. Downregulation of HKII decreased ATP production, which in turn further compromised hSPCA1 function that is greatly dependent on energy supply [26]. Our result showed that the level of HKII in the tissues of HHD patients were obviously decreased to about 60% of the normal value and demonstrates that increased Ca^2+^ concentration caused by *ATP2C1* mutation does result in decreased HKII, which reduces ATP production and affects normal physiological function of the human body.

## Conclusions

In this study, we detected four *ATP2C1* mutations in HHD patients, which has further enriched the pathogenic mutation pool of HHD. We showed that miR-203 was upregulated in HHD patients, which in turn might downregulate p63 and Notch1, and downregulation of p63 might further decrease the level of HKII. The exact mechanism underlying HHD pathogenesis remains unclear and needs further investigation.

## Data Availability

The datasets generated and/or analysed during the current study are available in the https://www.jianguoyun.com/p/DS_N9qkQ7Ym5CBjJ-ZkD. All data generated or analysed during this study can be easily acquired from corresponding author upon reasonable request.

## Abbreviations

HHD: Hailey-Hailey disease
hSPCA1: the human secretory pathway Ca^2+^/Mn^2+^ − ATPase protein 1
EDTA: Ethylene Diamine Tetracetic Acid
IR: inhibitor remover
WB: the rinse solution
HE: hematoxylin-eosin staining
PCR: Polymerase Chain Reaction
IHC: Immunohistochemistry
